# Letter from Dr. Joseph Clark of Dublin, to Dr. John Sims of London, Relative to Arm Presentation

**Published:** 1802-11-01

**Authors:** 


### *Letter from* Dr. Joseph Clark *of Dublin, to Dr.* John Sims *of London, relative to Arm Presentation.*

Sir,

I Have peruſed with ſatisfaction, your Obſervations "on Delivery in certain difficult Caſes of Arm Preſentation," contained in the 40th Number of the Medical and Phyſical Journal.

Much experience, in difficult caſes, ſuch as you deſcribe, had taught me to dread the operation of turning the fœtus in utero. Where the death of the fœtus is certain, either by the putrid ſtate of the integuments of the preſenting arm, or by a prolapsed umbilical chord, I have been accuſtomed ſome years paſt to inſtruct my pupils to attempt the operation of Embryotomy with Smellie's large perforating ſciſſars and crotchet, and to endeavour to get away the the fœtus piece-meal, in any manner which appeared to them leaſt diſtreſſing to the patient.

In July 1800, I was called on to deliberate with a ſurgeon of eminence in this city, in a caſe where the liquour amnii had been ſome days evacuated before any examination per vaginam was made; the arm and ſhoulder of a full grown putrid fœtus were impacted into the brim of the pelvis. Before I ſaw this patient, the gentleman in attendance had in vain attempted to turn the fœtus. The reſiſtance to every *reasonable* exertion appeared to him then, and to me, afterwards, inſurmountable. Under theſe circumſtances, I did not heſitate to propoſe that the preſenting arm ſhould be twiſted off, and the thorax perforated freely, with the view of diminiſhing the bulk of the preſenting part, and of promoting putrefaction. After theſe meaſures were put in practice, we agreed to wait the reſult of labour pains, which had hitherto been irregular and trivial. At the end of thirty-ſix hours the fœtus was expelled double. The patient's recovery was ſpeedy, and in every reſpect favourable.

The reſult of this ſingle caſe had determined me to make further trials of this practice, under ſimilar circumſtances; and the the perufal of your obfervations, leaves no room for doubting (in my mind) of the propriety of adopting it generally.

Should you think my teftimony on this fubject of any importance, I fhall not object to the publication of thefe remarks, however curfory, being fully perfuaded the practice propofed by you is well calculated to refcue the lives of our fellow creatures, in certain fituations, from the moft imminent danger.

*Dublin, Sept.* 87, 1802.

